# Sex-specific differences in the effect of the atherogenic index of plasma on prediabetes and diabetes in the NHANES 2011–2018 population

**DOI:** 10.1186/s12933-023-01740-8

**Published:** 2023-01-30

**Authors:** Yumeng Shi, Minghua Wen

**Affiliations:** 1 Department of Cardiovascular Medicine, The Second Affiliated Hospital of Nanchang University, No. 1 Minde Road, Nanchang, 330006 Jiangxi China; 2 Jiangxi Provincial Cardiovascular Disease Clinical Medical Research Center, Nanchang, Jiangxi China

**Keywords:** Atherogenic index of plasma, Prediabetes, Diabetes, Sex differences, Females

## Abstract

**Background:**

Although a great deal of scientific evidence on the epidemiological risk factors for diabetes and prediabetes has been accumulated, there is still insufficient evidence to explore sex-related differences. The aim of this study was to examine sex-specific differences in the effect of the atherogenic index of plasma (AIP) on prediabetes and diabetes.

**Methods:**

This cross-sectional study included data from 10099 American adults. The exposure variable was the AIP, which was defined as log10 (triglycerides/high-density lipoprotein cholesterol). The outcome variables included prediabetes and diabetes defined by the 2013 American Diabetes Association guidelines.

**Results:**

The median age (mean ± SD) was 48.51 ± 18.42 years, and the average value (SD) of the AIP was − 0.09 (0.34). The prevalence of prediabetes was 40.24%, and that of diabetes was 21.32%. Overall, there was a significant positive association between the AIP and prediabetes and diabetes (per 1-unit increment in the AIP: OR, 2.49; 95% CI 1.75, 3.54). The multivariate logistic regression model demonstrated that for each unit increment in the AIP, the prediabetes and diabetes prevalence increased 4.96-fold among female participants (OR 4.96, 95% CI 2.68, 9.18) but not among male participants. We found that the AIP was not related to the prevalence of prediabetes or diabetes (OR 1.41; 95% CI 0.87, 2.29) among males. There was an interaction between sex and the AIP (P for interaction < 0.0001).

**Conclusions:**

This study showed that a higher AIP was significantly associated with an increased prevalence of prediabetes and diabetes, and the above relationships occurred only among women and not men.

**Supplementary Information:**

The online version contains supplementary material available at 10.1186/s12933-023-01740-8.

## Introduction

In recent decades, type 2 diabetes (T2D), the most common and clinically important metabolic disease, has become a global epidemic and a major medical burden worldwide. According to the diabetes map released by the International Diabetes Federation (IDF), the number of adult diabetic patients worldwide reached 537 million in 2021, approximately 1 in 10 adults worldwide. The number of patients with diabetes increased by 74 million, or 16 percent, compared to 2019. It is estimated that the prevalence of diabetes will further increase to 12.2%, and the number of patients will increase to 783 million by 2025. Approximately 6.7 million people died from diabetes or diabetes complications in 2021, accounting for 12.2% of all deaths [1]. Prediabetes is defined as a concentration of blood glucose below the diabetes threshold but above the normal level, but it is related to the high risk of diabetes and its complications [2]. We must also pay attention to this disease state. More importantly, a large number of epidemiological studies have shown that in patients with prediabetes, the kidneys, blood vessels and heart are damaged [3, 4]. The physiological basis of the pathogenesis of diabetes or prediabetes is closely related to insulin resistance (IR) [5, 6]. Therefore, we should prevent and treat diabetes and prediabetes according to their pathological mechanisms to reduce the clinical complications related to diabetes and the occurrence and development of cardiovascular diseases (CVDs).

The atherogenic index of plasma (AIP) was first proposed by Dobiásová as a biomarker for plasma atherosclerosis [7]. An increasing number of studies have shown that the AIP is a powerful marker for predicting CVD risks [8–10]. nationwide population-based cohort study showed that the AIP was significantly associated with cardiovascular risks after adjusting for traditional risk factors [11], and was an independent factor for the prediction of developing cardiovascular events and their related mortality [12].The results of a prospective cohort study conducted by Zhang et al. among 5538 nondiabetic patients with coronary heart disease after percutaneous coronary intervention showed that compared with patients in the lower AIP group, patients in the higher AIP group had a 37% (HR: 1.37, 95% CI 1.04–1.81; p = 0.025) risk of major adverse cardiac events (MACEs), and the relationship between the hazard ratio and the AIP appeared to be J-shaped [13]. Moreover, the AIP is an independent predictive marker for rapid plaque progression beyond traditional risk factors [14] and might be a strong biomarker that could be used to predict the risk of cardiovascular events among patients with T2DM [15]. Moreover, studies have shown that the AIP is a predictor of diabetes [16–21]. However, no study has been conducted to explore the relationship between the AIP and prediabetes. In fact, the number of patients with prediabetes is much larger than the number of patients with diabetes [22]. Early detection of patients with prediabetes is of great importance for preventing their progression to diabetes.

Data from the National Health and Nutrition Examination Survey were used to conduct this cross-sectional study. The aim of this study was to examine sex-specific differences in the effect of the AIP on prediabetes and diabetes.

## Methods

### Study design and population

The data used in this study were all from the 2011–2018 National Health and Nutrition Examination Survey (NHANES) database. The NHANES is a continuous survey that selects a group of representative American people by means of complex and multistage probability sampling and aims to evaluate the health and nutrition status of American adults and children. The Ethics Review Committee of the National Center for Health Statistics (NCHS) approved the NHANES research plan. All the research participants provided written informed consent. More detailed information can be found at www.cdc.gov/nchs/nhanes/irba98.htm.

We conducted a cross-sectional study using data from the NHANES (2011–2018) study among patients ≥ 18 years of age (n = 10,978). The exclusion criterion was patients with missing AIP (n = 859), hemoglobin A1c or fasting plasma glucose (n = 20) data. Finally, 10099 subjects were analyzed.

### Definitions of the exposure and outcome variables

The exposure variable was the AIP, which was defined as log10 (triglycerides/high-density lipoprotein cholesterol) with triglycerides and high-density lipoprotein cholesterol expressed in mmol/L [16]. The outcome variables included prediabetes and diabetes. Prediabetes was defined as any one of the following: 5.7% ≤ hemoglobin A1c (HbA1c) < 6.5%, fasting plasma glucose (FPG) between 5.6 mmol/L and 7.0 mmol/L, and a 2 h FPG value between 7.8 mmol/L and 11.1 mmol/L during an oral glucose tolerance test (OGTT) in accordance with the 2013 American Diabetes Association guidelines [23].Diabetes was defined as a self-reported physician diagnosis of diabetes or having an HbA1c level ≥ 6.5%, FPG level ≥ 7 mmol/L, or 2 h OGTT plasma glucose level ≥ 11.1 mmol/L. The combination of prediabetes and diabetes was regarded as an end event in this analysis.

### Potential covariates

Covariables in this study included continuous variables (age, body mass index (BMI, kg/m2), systolic blood pressure (SBP, mmHg), diastolic blood pressure (DBP, mmHg), triglycerides (TGs, mg/dL), total cholesterol (TC, mg/dL), estimated glomerular filtration rate (eGFR, mL/min/1.73 m2), and poverty income ratio) and categorical variables (sex, race, smoking status, alcohol intake, antihypertensive drugs, and lipoprotein-lowering drugs). The interviews collected demographic information on age, sex (male or female), poverty income ratio, race (non-Hispanic white, non-Hispanic black, Mexican American, other Hispanic, or other), smoking status (never, former, or current), alcohol intake (< 3, ≥ 3 drinks per day), antihypertensive drugs (no, yes), and lipoprotein-lowering drugs (no, yes). Anthropometric indicators included height, weight and blood pressure (BP). Body height and weight were collected without shoes and in light clothing and measured with a medical scale. Body mass index (BMI) in kg/m^2^ was calculated as weight divided by height squared. According to the standard blood pressure measurement protocol recommended by the American Heart Association at that time, a mercury sphygmomanometer was used to measure blood pressure. Three blood pressure readings were obtained continuously from the same arm. This study defined SBP and DBP as the average of three blood pressure measurements. Every subject was asked to provide an overnight rapid venous blood sample.

Fasting venous blood was drawn from each subject for TC and TG measurement. The formula used for the estimated glomerular filtration rate (eGFR) was the Chronic Kidney Disease Epidemiology Collaboration (CKD-EPI) equation [24].

### Statistical analysis

The statistical analyses of this study were performed via R, version 4.2.0 (R Foundation) and EmpowerStats (http://www.empowerstats.com, X&Y Solutions, Inc., Boston, MA). The level of statistical significance was set at p < 0.05.

Baseline characteristics are presented as the means and standard deviations (SDs) or medians (interquartile ranges) (IQRs) for continuous variables and proportions for categorical variables. The quartiles of AIP groups were compared with Student’s t test, the Mann–Whitney U test, and the chi-square test for categorical variables. Covariates that were known to be traditional or suspected risk factors for prediabetes and diabetes or the estimates of the AIP on prediabetes and diabetes changed by more than 10% in the multivariate logistic regression models [25]. The relationship between the AIP and prediabetes and diabetes in the overall population and among males and females was investigated with multivariate logistic regression analysis. Model 1 represented the unadjusted data. In Model 2, the data were adjusted for age, sex (only for the overall population), BMI, race, SBP, DBP, TGs, and TC. In Model 3, the results were adjusted for age, sex (only for the overall population), BMI, race, SBP, DBP, TGs, TC, eGFR, poverty income ratio, current smoking, alcohol intake, antihypertensive drugs, and lipoprotein-lowering drugs. The results from the logistic regression analysis are presented as odds ratios (ORs) and 95% confidence intervals (CIs). Moreover, the effect dose response between the AIP and prediabetes and diabetes was evaluated by a generalized additive model and fitting curve (penalized spline method). The interaction of the AIP with different sexes on prediabetes and diabetes was assessed by including stratification analysis and interaction tests in the regression model.

## Results

There were 10,099 participants, including 4913 males and 5186 females. The median age (mean ± SD) was 48.51 ± 18.42 years, and the average value (SD) of the AIP was − 0.09 (0.34). The prevalence of prediabetes was 40.24%, and that of diabetes was 21.32%. Table 1 shows participant demographic and clinical characteristics by quartiles of the baseline AIP. In the four AIP groups, all variables were statistically significant. Compared with the participants in the lower AIP group, the participants in the AIP Q4 group were often male, older, non-Hispanic white, current smokers and drinkers, had higher levels of SBP, DBP, TC, and TGs, had a lower poverty income ratio and eGFR, and had a higher proportion of antihypertensive drug and lipid-lowering drug use.

**Table 1:** The demographic and clinical characteristics of the patients by quartiles of baseline AIP

Variable^a^	AIP Quartiles				*P* value
	Q1(< − 0.32)	Q2(− 0.32 to < − 0.10)	Q3(− 0.10 to < 0.12)	Q4(≥ 0.12)	
Participants	2522	2524	2528	2525	
Males, N (%)	916 (36.32%)	1123 (44.49%)	1292 (51.11%)	1582 (62.65%)	< 0.001
Age,year	45.28 ± 19.34	47.96 ± 18.91	50.70 ± 18.13	50.11 ± 16.69	< 0.001
BMI, kg/m^2^	26.33 ± 6.57	28.31 ± 6.91	30.18 ± 7.05	31.61 ± 6.83	< 0.001
Race					< 0.001
Non-Hispanic White, N (%)	858 (34.02%)	900 (35.66%)	948 (37.50%)	1084 (42.93%)	
Non-Hispanic Black, N (%)	821 (32.55%)	640 (25.36%)	437 (17.29%)	276 (10.93%)	
Mexican American, N (%)	216 (8.56%)	329 (13.03%)	405 (16.02%)	447 (17.70%)	
Other Hispanic, N (%)	195 (7.73%)	244 (9.67%)	314 (12.42%)	319 (12.63%)	
Other races, N (%)	432 (17.13%)	411 (16.28%)	424 (16.77%)	399 (15.80%)	
Current smoking, N (%)	508 (20.46%)	528 (21.26%)	602 (24.16%)	678 (27.06%)	< 0.001
alcohol intake,drinks per day					< 0.001
< 3	1124 (69.25%)	1019 (65.74%)	939 (61.41%)	907 (59.17%)	
≥ 3	499 (30.75%)	531 (34.26%)	590 (38.59%)	626 (40.83%)	
SBP, mmHg	120.27 ± 18.54	122.95 ± 19.13	125.28 ± 18.91	125.81 ± 17.55	< 0.001
DBP, mmHg	68.21 ± 11.32	68.94 ± 12.06	70.29 ± 11.72	71.90 ± 12.01	< 0.001
Poverty income ratio	2.58 ± 1.67	2.47 ± 1.64	2.40 ± 1.63	2.32 ± 1.57	< 0.001
TC, mg/dL	179.59 ± 37.78	183.15 ± 37.80	188.26 ± 41.93	201.16 ± 45.18	< 0.001
TG, mg/dL	51.00 (41.00–61.00)	79.00 (68.00–92.00)	112.00 (98.00–129.00)	187.00 (154.00–244.00)	< 0.001
eGFR, mL/min/1.73 m^2^	101.11 ± 24.31	96.83 ± 24.26	93.95 ± 24.08	92.71 ± 24.15	< 0.001
Glucose metabolism state					< 0.001
none prediabetes	1398 (55.43%)	1100 (43.58%)	811 (32.08%)	573 (22.69%)	
prediabetes	865 (34.30%)	1021 (40.45%)	1077 (42.60%)	1101 (43.60%)	
diabetes	259 (10.27%)	403 (15.97%)	640 (25.32%)	851 (33.70%)	
Antihypertensive drugs	104 (4.12%)	144 (5.71%)	171 (6.76%)	166 (6.57%)	< 0.001
Lipoprotein-lowering drugs	352 (13.96%)	497 (19.69%)	624 (24.68%)	660 (26.14%)	< 0.001

*AIP* atherogenic index of plasma, *BMI* body mass index, *SBP* systolic blood pressure, *DBP* diastolic blood pressure, *TC* total cholesterol, *TG* triglycerides, *eGFR* estimated glomerular filtration rate

^a^Data are presented as number (%) or mean ± standard deviation

**Fig. 1:**
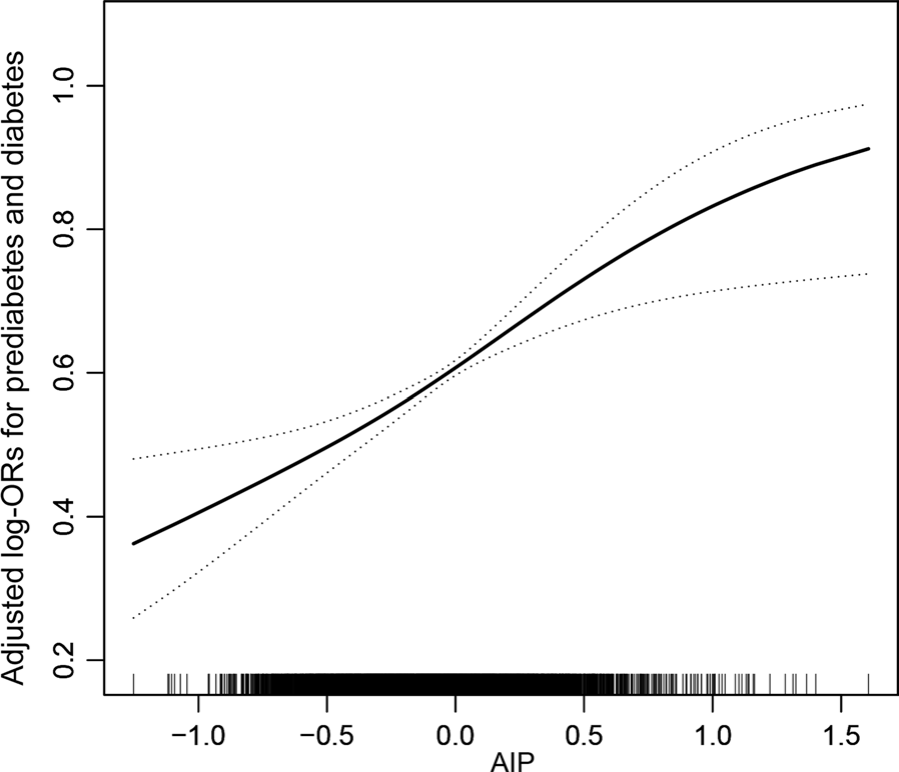
Association between AIP and the prevalence of prediabetes and diabetes. A linear association between AIP and the prevalence of prediabetes and diabetes was found (P < 0.05). The solid line and dashed line represent the estimated values and their corresponding 95% confidence interval. Adjustment factors included age, sex, BMI, race, SBP, DBP, TG, TC, eGFR, poverty income ratio, current smoking, alcohol intake, antihypertensive drugs, lipoprotein-lowering drugs

Regardless of whether the confounding factors were adjusted for, the AIP had a significant positive correlation with prediabetes and diabetes among all participants. The AIP was further divided into quartiles, and the Q1 group was used as the reference group to evaluate the relationship between the AIP and prediabetes and diabetes. After adjusting for age, sex, BMI, race, SBP, DBP, TGs, TC, eGFR, poverty income ratio, current smoking, alcohol intake, antihypertensive drugs, and lipoprotein-lowering drugs, compared with the Q1 reference group, the relative odds of prediabetes and diabetes of the participants in the Q2 (OR: 1.16; 95%: 0.97, 1.39), Q3 (OR: 1.46; 95%: 1.20, 1.78) and Q4 (OR: 1.95; 95%: 1.50, 2.55) groups increased linearly. With a P for trend < 0.0001, the AIP had a linear positive correlation with prediabetes and diabetes (Table 2). The results of multivariate regression analysis were consistent with those of the fitting curve (Fig. 1).

**Table 2:** Relative odds of prediabetes and diabetes according to AIP in different models among all participants^a^

AIP	Events (%)	prediabetes and diabetes *OR* (95%CI)		
		Model 1	Model 2	Model 3
Per 1 increment	6217 (61.56%)	5.46 (4.77, 6.25)***	2.85 (2.18, 3.74)***	2.49 (1.75, 3.54)***
Quartile				
Q1(< − 0.32)	1124 (44.57%)	1	1	1
Q2(-0.32 to < − 0.10)	1424 (56.42%)	1.61 (1.44, 1.80) ***	1.25 (1.09, 1.43)**	1.16 (0.97, 1.39)
Q3(− 0.10 to < 0.12)	1717 (67.92%)	2.63 (2.35, 2.95) ***	1.57 (1.35, 1.82)***	1.46 (1.20, 1.78)***
Q4(≥ 0.12)	1952 (77.31%)	4.24 (3.75, 4.79) ***	2.10 (1.70, 2.59)***	1.95 (1.50, 2.55)***
*P for trend*		< 0.0001	< 0.0001	< 0.0001

^a^Values are ORs (95% CIs) unless otherwise indicated

*AIP* Atherogenic index of plasma

^*^P < 0.05

^**^P < 0.01

^***^P < 0.001

Model 1 was adjusted for none

Model 2 was adjusted for age, sex, BMI, race, SBP, DBP, TG, TC

Model 3was adjusted for age, sex, BMI, race, SBP, DBP, TG, TC, eGFR, poverty income ratio, current smoking, alcohol intake, antihypertensive drugs, lipoprotein-lowering drugs

**Fig. 2:**
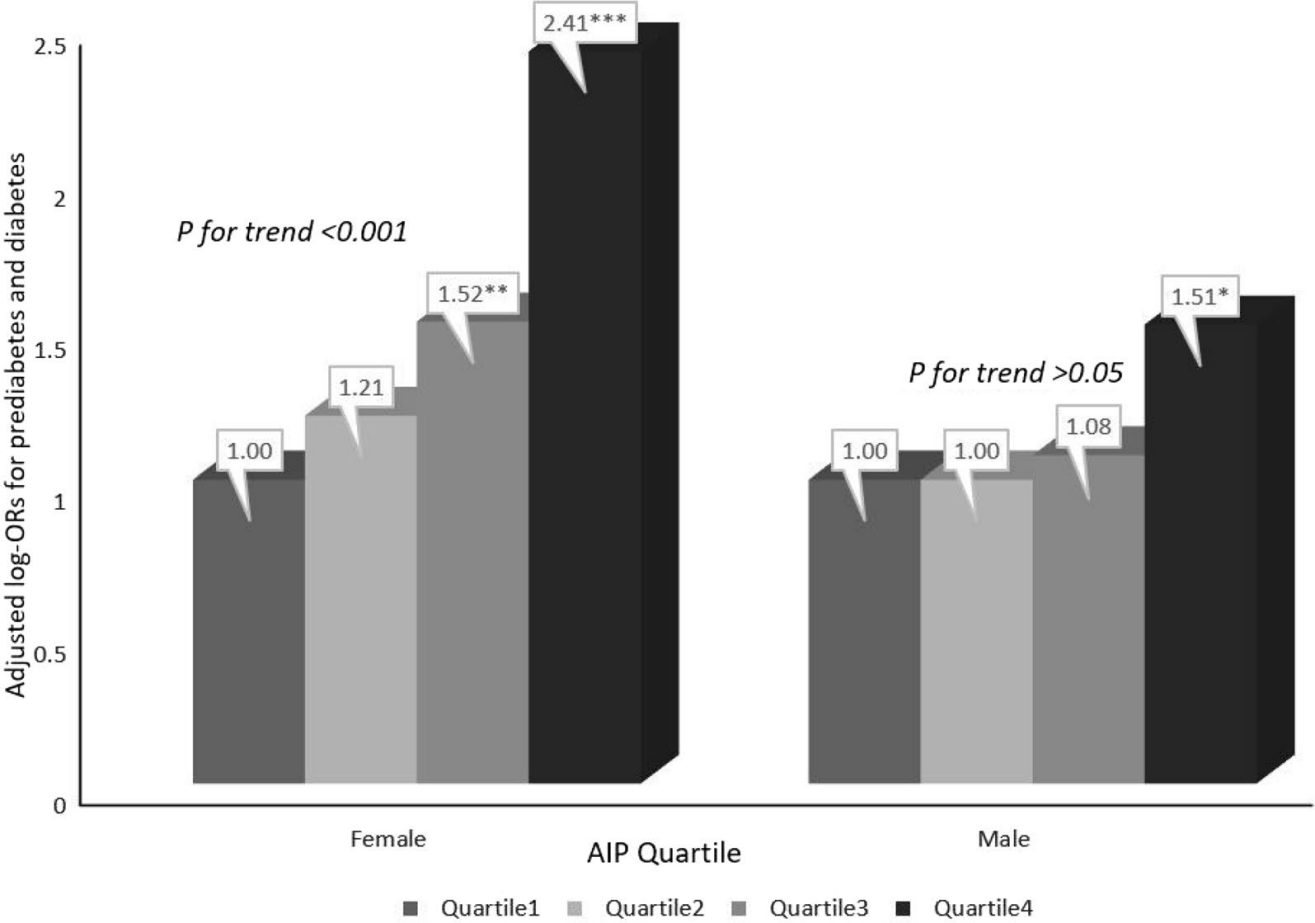
Relative odds of prediabetes and diabetes according to AIP quartile among male and female^#^.^#^The adjustment factors included age, BMI, race, SBP, DBP, TG, TC, eGFR, poverty income ratio, current smoking, alcohol intake, antihypertensive drugs, lipoprotein-lowering drugs. *P<0.05 **P<0.01 ***P<0.001

Table 3 shows the relationship between the AIP and prediabetes and diabetes among men and women; sex differences were found. With the increase in the AIP in three different models, the risk of prediabetes and diabetes among women was more significant than that among men (P for interaction < 0.0001). In addition, in fully adjusted Model 3, the AIP of male participants was not related to prediabetes or diabetes. Likewise, in addition to the AIP quartiles by sex, the influence of the AIP as a categorical variable on prediabetes and diabetes was analyzed. A multivariate regression analysis of the AIP, prediabetes and diabetes for the different sexes is shown in Fig. 2. Compared with the Q1 reference group, the relative odds of prediabetes and diabetes of the participants in the Q2 (OR: 1.21; 95%: 0.93, 1.58), Q3 (OR: 1.52; 95%: 1.11, 2.07) and Q4 (OR: 2.41; 95%: 1.53, 3.79) groups gradually increased among females (P for trend < 0.0001). However, we found that the fully adjusted Model 3 AIP for men was not related to prediabetes or diabetes, regardless of whether the AIP was a continuous variable or a categorical variable (P for trend = 0.055).

**Table 3:** Relative odds of prediabetes and diabetes according to AIP in different models among male and female

AIP	Events (%)	prediabetes and diabetes *OR* (95%CI)		
		Model 1	Model 2	Model 3
Male				
Per 1 increment	3274 (66.64%)	3.20 (2.66, 3.86)***	1.72 (1.17, 2.53)**	1.41 (0.87, 2.29)
Q1(< − 0.25)	695 (56.64%)	1	1	1
Q2(−0.25 to < -0.03)	762 (62.00%)	1.25 (1.06, 1.47)**	0.97 (0.80, 1.17)	1.00 (0.78, 1.27)
Q3(− 0.03 to < 0.20)	856 (69.71%)	1.76 (1.49, 2.08)***	1.19 (0.97, 1.47)	1.08 (0.83, 1.40)
Q4(≥ 0.02)	961 (78.19%)	2.74 (2.30, 3.27)***	1.59 (1.20, 2.12)**	1.51 (1.07, 2.14)*
*P for trend*		< 0.0001	0.0022	0.055
Female				
Per 1 increment	2943 (56.75%)	8.47 (6.90, 10.39) ***	4.26 (2.68, 6.78)***	4.96 (2.68, 9.18)***
Q1(< − 0.37)	481 (37.09%)	1	1	1
Q2(− 0.37 to < -0.17)	652 (50.31%)	1.72 (1.47, 2.01)***	1.33 (1.10, 1.61)**	1.21 (0.93, 1.58)
Q3(− 0.17 to < 0.05)	825 (63.66%)	2.97 (2.53, 3.49)***	1.64 (1.31, 2.05)***	1.52 (1.11, 2.07)**
Q4(≥ 0.05)	985 (75.94%)	5.36 (4.52, 6.35)***	2.23 (1.60, 3.10)***	2.41 (1.53, 3.79)***
*P for trend*		< 0.0001	< 0.0001	0.0003
P value for interaction*		< 0.0001	< 0.0001	< 0.0001

^*^P < 0.05

^**^P < 0.01

^***^P < 0.001

Model 1 was adjusted for none

Model 2 was adjusted for age, BMI, race, SBP, DBP, TG, TC

Model 3 was adjusted for age, BMI, race, SBP, DBP, TG, TC, eGFR, poverty income ratio, current smoking, alcohol intake, antihypertensive drugs, lipoprotein-lowering drugs

## Discussion

This was a large-sample cross-sectional study based on 2011–2018 National Health and Nutrition Survey data that found that an increased AIP increased the risk of prediabetes and diabetes. Further analysis found that an excessive AIP only increased the risk of prediabetes and diabetes among women. This is the first study to evaluate the relationship between sex differences in the AIP and prediabetes and diabetes.

Previous studies have evaluated the relationship between the AIP and diabetes, and all of them have shown a positive correlation between the AIP and diabetes [16–20]. A meta-analysis of 15 case–control studies showed that the ability of the AIP to predict the risk of diabetes was better than that of other lipid components [21]. Li and colleagues conducted a cohort study in the general population of Taiwan Province among 7670 individuals to assess the relationship between AIP levels and diabetes risk among participants of different ages and sexes. The results showed that only participants aged 40–64 years had a higher diabetes risk, and no sex differences were found [20]. However, we did not find any age differences in our research. We divided age by 20-year categories. The AIP was positively correlated with prediabetes and diabetes in each age group, but it did not reach statistical significance because there were too few people under 20 and over 80 years of age (see Additional file 1: Table S1 for specific results). A total of 2676 individuals aged 28–80 years in the general population were selected for the Turkish Adult Risk Factor Study, and in the observation of the relationship between the AIP and hypertension, diabetes and metabolic syndrome, the final result showed that the AIP had an independent positive correlation with diabetes among both men and women [18].The cross-sectional study found significant sex differences, which may be due to the different characteristics of the study participants.

In fact, there are more patients with prediabetes, and prediabetes easily develops into diabetes. Therefore, in this study, we used the combination of prediabetes and diabetes as an outcome variable for the first time to observe the relationship between the AIP and both prediabetes and diabetes. This cross-sectional analysis found that the AIP had a significant linear positive correlation with prediabetes and diabetes, regardless of whether confounding factors were adjusted for. Compared with the lowest AIP group, the risk of prediabetes and diabetes in the highest AIP group increased significantly by 95%. We further evaluated the relationship between the AIP and prediabetes and diabetes by sex and found that there was a linear positive correlation between the AIP and outcome events among females but not among males. One accepted hypothesis is that the higher risk of diabetes among women in late adulthood is due to hormonal changes in menopause, that is, estrogen consumption [26, 27]. Moreover, there are also epidemiological studies that show that the risk of diabetes and its related complications is higher among women than among men as they age [28, 29].

The mechanism of the AIP in prediabetes and diabetes is not clear, but the following biological mechanisms can be explained. The AIP is calculated by combining TGs and HDL, so the levels of TGs and HDL in the human body are closely related to the pathogenesis of diabetes and prediabetes. High levels of TGs in plasma reduce the number and activity of insulin receptors on adipocytes and prevent insulin from binding to receptors by competing with glucose to enter cells, leading to diabetes, while lower HDL levels also lead to decreased insulin secretion and sensitivity [30].Abnormal blood lipid levels may cause insulin resistance (IR) by causing inflammation, endoplasmic reticulum stress and lipotoxicity [31]. Moreover, IR increases TG and plasma-free fatty acid levels, while HDL-C levels are decreased. Both are causal, which verifies the "vicious circle" hypothesis of diabetes development. As an early state of diabetes, prediabetes has the same risk factors as diabetes [32]. Some studies have shown that there are metabolic abnormalities in prediabetic patients, such as abnormal blood glucose levels, dyslipidemia, IR, a procoagulant state, endothelial dysfunction, oxidative stress and inflammation [33]. Therefore, the causes of prediabetes and diabetes by the AIP are still based on IR and dyslipidemia.

## Limitations

There are some shortcomings in this study. First, this was a cross-sectional study, a design that is not as comprehensive as a cohort study. In addition, this study’s ability to explore the etiology hypothesis was limited, and its ability to test the etiology hypothesis and extrapolation was not sufficient. Other cohort studies are needed to verify the correlation between the AIP and prediabetes and diabetes. Second, confounding by unknown or unmeasurable factors could not be completely ruled out. Finally, due to the differences between countries, the results may not be extrapolated to other countries.

## Perspectives and significance section

Sex differences exist throughout the life cycle, but their specific mechanisms and consequences are still unclear. Knowing the sex differences in risk factors for diabetes and prediabetes can help clinicians implement more personalized prevention strategies correctly.

## Conclusions

This cross-sectional study was conducted among 10,099 American adults and showed a positive correlation between the AIP and prediabetes and diabetes, and the risk of prediabetes and diabetes increased gradually with the increase in the AIP. Moreover, we found an interaction between sex and the AIP and found that the AIP was positively correlated with prediabetes and diabetes only among women and not among men.

## Supplementary Information

**Additional file1: Table S1.** Relative odds of prediabetes and diabetes according to AIP in different ages.

## Data Availability

Publicly available datasets were analyzed in this study. This data can be found here: https://www.cdc.gov/nchs/nhanes/index.htm.
