# Supplementary material for: Sex-specific differences in the effect of the atherogenic index of plasma on prediabetes and diabetes in the NHANES 2011–2018 population
Source: Cardiovasc Diabetol. 2023 Jan 30;22:19. doi: 10.1186/s12933-023-01740-8 (PMC9887826; doi:10.1186/s12933-023-01740-8)
Supplement: Supplementary file 1 — Additional file1: Table S1. Relative odds of prediabetes and diabetes according to AIP in different ages. [file 12933_2023_1740_MOESM1_ESM.docx]

Additional file 1: Table S1. Relative odds of prediabetes and diabetes according to AIP in different ages^1^

| AIP (Per 1 increment) | Event(%) | prediabetes and diabetes, OR (95%CI) | P for interaction |
| --- | --- | --- | --- |
| Age,years |  |  | 0.538 |
| ＜20 | 136 (28.04%) | 2.04 (0.55, 7.60) |  |
| 20 to<40 | 1200 (38.68%) | 2.10 (1.36, 3.24)*** |  |
| 40 to<60 | 2173 (67.40%) | 3.05 (1.93, 4.82)*** |  |
| 60 to<80 | 2212 (82.11%) | 2.48 (1.43, 4.29)** |  |
| ≥80 | 496 (83.50%) | 3.76 (0.91, 15.50) |  |

^1^Values are ORs (95% CIs) unless otherwise indicated. AIP, Atherogenic index of plasma.

**P<0.01, ***P<0.001

The adjustment factors included age, sex, BMI, race, SBP, DBP, TG, TC, eGFR, poverty income ratio, current smoking, alcohol intake, antihypertensive drugs, lipoprotein-lowering drugs.
